# Explainable and High-Performance ECG-Informed Machine Learning and Deep Learning Framework for Cardiovascular Risk Prediction

**DOI:** 10.3390/life16081259

**Published:** 2026-07-30

**Authors:** Suliman Aladhadh

**Affiliations:** Department of Information Technology, College of Computer, Qassim University, Buraydah 52571, Saudi Arabia; s.aladhadh@qu.edu.sa

**Keywords:** cardiovascular disease risk prediction, electrocardiogram (ECG), explainable artificial intelligence, machine learning, deep learning, SHAP, clinical decision support

## Abstract

Predictive models used for the assessment of cardiovascular disease (CVD) risk must not only be highly accurate but also explainable and reliable for clinical decision-making. In this paper, we present a methodological framework for explainable machine learning and deep learning approaches to CVD risk prediction that uses clinically derived electrocardiogram (ECG) features and conventional CVD risk factors. A large synthetic dataset containing 30,000 patient cases was created according to a clinically validated distribution to provide reproducible and privacy-preserving benchmarking. A series of interpretability tests were performed on multiple models of machine learning and deep learning categories within a unified experimental setup. Out of the tested models, XGBoost and BiLSTM provided the highest discrimination capabilities in the machine learning and deep learning categories, respectively. Explaining the predictions via the SHapley Additive explanations (SHAP) approach, calibration of probability estimations, uncertainty quantification, and ablation studies were applied to assess the explainability, reliability, and robustness of the models. We show that the presented framework allows for the production of consistent and interpretable predictions along with the evaluation of the models’ explainability and predictive reliability. Instead of focusing on the single-feature importance, this paper presents a reproducible methodological framework for the explainable modeling of CVD risk.

## 1. Introduction

CVDs remain one of the major health concerns across the world, accounting for a high percentage of morbidity and mortality [1]. Therefore, early and precise assessment of cardiovascular risk is important for both preventive interventions and clinical decision-making. In recent years, the increasing availability of digital health records and physiological signals has made data-driven approaches using machine learning and deep learning promising tools for automated CVD risk prediction [2,3].

The ECG is one of the most frequently utilized, non-invasive diagnostic modalities [4]. It illustrates the electrical activity of the heart and provides critical information related to arrhythmias, ischemia, and structural abnormalities. Although ECGs are obtained as a routine check-up in clinical practice, their use in computational CVD risk prediction remains very limited [5,6]. Classic risk models depend mainly on demographic and laboratory-based factors, whereas most recent deep learning methods process raw ECG signals using end-to-end architectures that have limited interpretability. This lack of transparency reduces clinical confidence and hinders practical deployment in real-world healthcare systems [7].

Explainability, calibration, and robustness are some of the most fundamental requirements associated with AI-driven clinical decision support systems. However, many existing studies focus primarily on predictive performance alone, with a very limited systematic evaluation of model interpretability, probability reliability, and feature-level contributions. Furthermore, research in this domain is greatly constrained by limited access to large, well-annotated ECG datasets due to privacy and regulatory restrictions that hinder reproducibility and comparative evaluation [8,9].

To tackle these challenges, this research suggests a new framework using explainable machine learning and deep learning techniques that can model cardiovascular risk using both ECG-derived characteristics as well as conventional cardiovascular risk factors. The framework conducts a comparative analysis of several explainable machine learning and deep learning models, along with the use of SHapley Additive explanations (SHAP) explainability, probability calibration, uncertainty estimation, and feature ablation.

To address these challenges, this paper presents an explainable machine learning and deep learning framework for cardiovascular risk prediction using ECG-dominant features combined with minimal clinical risk factors. A large-scale synthetic dataset was constructed based on clinically validated distributions and risk relationships as reported in established ECG repositories and cardiovascular epidemiological studies. This dataset facilitates controlled experimentation, reproducible benchmarking, and in-depth explainability without compromising patient privacy.

A systematic review of interpretable machine learning models and deep learning architectures was conducted within a unified experimental framework. Model performance was evaluated through a set of standard metrics in classification problems and receiver operating characteristic analysis. The interpretability of models relies on SHAP-based feature attribution. Moreover, experiments regarding probability calibration, uncertainty quantification, and feature ablation were conducted to evaluate model reliability and robustness. Of special note is the quantification of the contribution of ECG-derived features relative to conventional cardiovascular risk factors.

The electrocardiogram (ECG)-informed models were developed and benchmarked, using both classical and deep learning approaches, to achieve the following aims: (i) to develop and benchmark ECG-informed CVD risk prediction models using classical and deep learning; (ii) to provide transparent and clinically meaningful explanations of model predictions; and (iii) to evaluate calibration, uncertainty, and robustness to support reliable risk estimation. This study makes several contributions, including an ECG-focused synthetic cardiovascular dataset, a comprehensive comparative modeling analysis, and an explainability-driven evaluation framework supporting trustworthy AI development for cardiovascular risk assessment.

The rest of the paper is organized as follows: Section 2 presents the literature review, Section 3 discusses the overall research design and procedure, Section 4 discusses the experimental results, and finally, Section 5 concludes the study.

## 2. Literature Review

The integration of artificial intelligence (AI) and machine learning (ML) has experienced significant growth in various sectors, with the medical field, particularly in the prediction of heart-related diseases, being one of the rapidly emerging areas of interest over the last decade [10]. Although significant research and development have been conducted on ML models for heart disease prediction, many existing models lack novelty. Analysis of existing research indicates that artificial neural networks (ANNs), support vector machines (SVMs), decision trees, and random forests have been thoroughly utilized for this purpose [11]. Bouqentar et al. [12] obtained 95% accuracy with ANN models, while existing research indicates a potential accuracy of up to 99% using random forest approaches [13]. Moreover, CNNs have demonstrated significant forecast accuracy on extensive ECG datasets, and recent studies have broken the 94% accuracy barrier [14].

Talin et al. [15] integrated classical statistical analysis with machine learning techniques to reveal key predictors of heart disease, including chest pain, the number of large arteries affected, and thalassemia. By using SHAP values together with Borda count rankings of various ML algorithms, these authors proved the benefit of combining explainability and statistical strength to increase accuracy rates of diagnosis. The XAI initiative has further met the need for explainability of complex ML algorithms, and particularly, their application in healthcare environments, which raises important concerns about the transparency of ongoing computations and intervention of AI systems in healthcare delivery processes. Recently, a survey has described and guided efforts to apply various interpretability means, underlining their relevance in healthcare environments, and stressing the transparency of clinical decision-making processes [16]. CNNs together with deep SHAP analysis have further been utilized to increase diagnostic performance and transparency of heart disease, indicating a great promise in integrating deep learning with explainable analysis tools [17].

Moreover, more recent works have applied sophisticated ensemble and boosting methods such as gradient boosting, voting, and stacking classifiers, with prediction accuracies over 91% to predict the mortality of heart failure. These works applied SHAP analysis and dealt with the class imbalance problem using techniques such as SMOTE and bootstrapping, and pinpointed important predictors like time, serum creatinine, and ejection fraction, among others [18]. Notably, the above-mentioned works demonstrate the growing demand for explainable ML models and are relevant to the current study, with its aim to explore explainable predictive models that are relevant to clinicians.

Moving further with the aid of advancements in the application of artificial intelligence, the significance of Explainable AI has appeared, especially in the region of healthcare, where the aspects of transparency and interpretability assume great importance for adoption [19,20]. Some techniques have been developed for the “black box” problem that arises with the complex implementation of ML models [21]. SHAP (SHapley Additive Explanations), for example, has used the principles of game theory for the interpretation of feature importance scores, explained both globally as well as locally, but with the possibility of increased computations [21]. LIME (Local Interpretable Model-agnostic Explanations) can also explain individual predictions using the technique of local surrogate models, but with the possibility of varying interpretations for similar situations [22]. SHAP and LIME are post-learning strategies and have post-training applications with the ability to explain the model, but with the possibility of inducing increased computations [23].

Explainable Boosting Machines (EBMs) can be classified under the wider category of explainable approaches to ML, ensuring the integration of the forecasting capabilities of boosters with the model explainability altogether. Unlike other explanation methods, EBMs remain inherently interpretable, making clinical usage more feasible and decision-making more credible [21]. With the capacity to integrate accuracy with the model’s explainability, EBMs automatically overcome the need to be deeply explained after the training phase is completed.

A generalized additive model with pairwise interactions is the primary platform for EBMs, allowing the individual contribution to be graphically shown for each feature. This is better aligned with the clinical reasoning process. Computational simplicity with the capacity to offer explanations at the global or local scale together makes EBMs more appropriate for clinical usage, where rapid decision-making is required, along with the need for regulatory compliance. For the particular case of myocardial infarction, EBMs are capable of specifying the important biochemical variables associated with cardiovascular risk with accuracy, also pointing out the rank and extent of the influence exerted by each feature clearly, which is more interpretable for the clinical professional.

### Research Gap

Despite these developments, many limitations are still associated with current research: most studies rely on small or private datasets; there is no systematic integration of ECG-derived features with minimal yet clinically relevant risk factors; and there is a high dependence on post hoc explainability techniques, which may introduce variability and add computational overhead. Moreover, only a few of them carefully assess model calibration, uncertainty, and robustness, thus limiting the clinical applicability of existing approaches. In particular, the potential of inherently interpretable models, such as EBMs, has not been explored for ECG-informed cardiovascular risk prediction. This work tries to fill these gaps by developing a complete, reproducible framework that fuses ECG-dominant features with essential clinical variables, benchmarks both classical and deep learning approaches, and provides a systematic evaluation of interpretability, calibration, and robustness, thereby offering a clinically trustworthy basis for cardiovascular risk assessment.

## 3. Research Design

**Figure 1:**
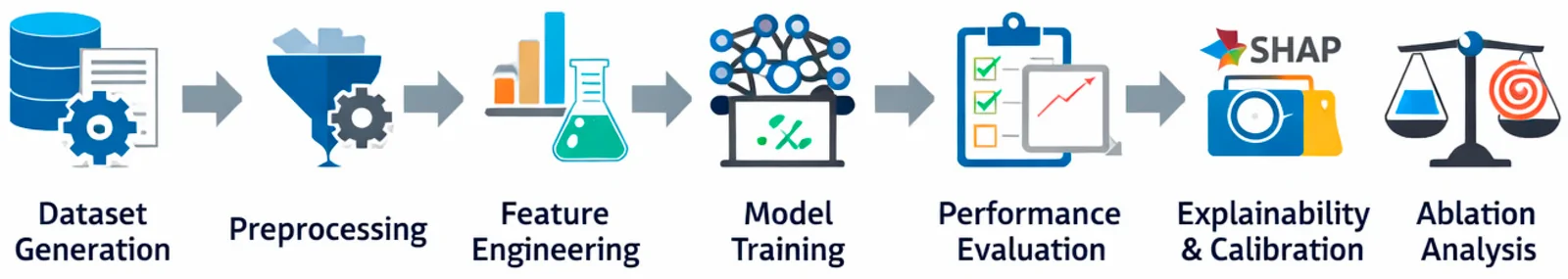
Overall methodological pipeline.

The current study embraces a quantitative, experimental research design in order to develop and evaluate explainable models of machine learning and deep learning for CVD risk prediction using ECG-dominant features complemented by minimal clinical risk factors. In general, the workflow involves synthetic dataset generation, data preprocessing, feature standardization, model training and testing, performance evaluation, explainability analysis, calibration assessment, and robustness validation via ablation experiments. Figure 1 shows a conceptual view of the methodological pipeline. To ensure clarity and consistency in our experiments and to make them easily reproducible, all experiments presented in this research were carried out on a single data configuration: one synthetic dataset of 30,000 records with an 8.0% high-risk prevalence and an 80:20 stratified train/test split. There are no experiments using subsampled data or smaller data sizes in the rest of this research.

### 3.1. Dataset Description

To support explainable cardiovascular risk prediction, 30,000 artificial patient records were created. The data is in the form of a comma-separated values file, synthetic-ecg-cvd-dataset.csv. The artificially generated data do not have any data gaps and can thus be directly used in machine learning or statistical modeling without imputation. Clinically validated distributions and risk relationships in the literature and based on existing cardiovascular and ECG data were used to synthesize data, including the PTB-XL ECG dataset, MIT-BIH Arrhythmia Database, Framingham Heart Study, and American Heart Association guidelines. The marginal distributions, inter-feature associations, and risk associations were guided by these sources to make the distributions clinically plausible without infringing on patient privacy. The dataset focuses on the central physiological characteristics of ECG as its first contribution, with a minimal but clinically significant amount of demographic and cardiovascular risk factors. Records belong to each separate patient with demographic characteristics, cardiovascular risk profiles, ECG-based parameters, and cardiovascular risk labels. The target variable consists of a continuous cardiovascular risk score and a derived binary cardiovascular disease (CVD) risk label defined as:

(1) $$y=\{\begin{array}{cc} 1, & \mathrm{if}\;R\geq T\\ 0, & \mathrm{if}\;R<T \end{array}$$

where R denotes the continuous synthetic cardiovascular risk score and T represents a clinically motivated decision threshold used to distinguish low-risk from high-risk individuals. In this study, the threshold value was empirically chosen during the generation of the dataset to obtain a clinically plausible rate of around 8.0% high-risk subjects. Instead of applying direct labels, a risk score was computed based on clinically driven relations between demographic, clinical, and ECG variables, which were later binarized by applying a threshold. This method is more representative of the way clinical risk assessments are done, where risk scores are usually binarized into categorical groups.

### 3.2. Synthetic Data Generation and Reproducibility

The synthetic cardiovascular cohort was produced by using a completely deterministic, rule-driven simulation pipeline that is guaranteed to reproduce exactly those marginal distributions, dependencies between variables, and physiological constraints that have been clinically validated. For that purpose, all random processes were initialized with a predefined random seed set to 42.

Demographic and conventional clinical variables were sampled using parametric data distributions based on large epidemiological surveys. For example, age was sampled from a truncated normal distribution with parameters set to mean = 55 years, standard deviation = 12 years, and range defined over the interval from 18 to 90 years, based on demographic data provided in the Framingham Heart Study. The variable sex was sampled from a Bernoulli distribution with a probability of 0.52 assigned to male. Systolic and diastolic BP were sampled from age- and sex-stratified normal distributions, defined over reference values provided in the American Heart Association recommendations. Systolic BP was constrained to be both higher than diastolic BP and to fall in the range from 90 to 200 mmHg. Similar logic was used to sample lipid values (total cholesterol, LDL, HDL) from correlated Gaussian distributions derived from Framingham and NHANES statistical surveys.

Binary clinical risk factors, for instance, diabetes, smoking status, and family history of cardiovascular disease, were also created as an outcome of Bernoulli trials with probabilities conditionally dependent on age groups and sex. For example, the prevalence of diabetes increased monotonically with age groups. The probabilities of smoking were higher in younger and middle-aged adults.

ECG-related features are generated synthetically to represent summary statistics rather than individual waveform traces. Such feature values and correlations are based on ECG summary statistics from PTB-XL and MIT-BIH Arrhythmia Database. Heart rates are sampled from a normal distribution with a mean of 72 beats/min and a standard deviation of 10. Heart rates are mildly positively correlated with age and systolic BP. QT duration and QTc are based on sex-specific baseline distributions. QTc is adjusted according to Bazett’s formula while ensuring all QTc intervals are within clinically acceptable ranges. The amplitude of elevation in ST segments follows a uniform continuous distribution centered near zero with minimal variance. Occasional large values are introduced by applying probabilistic rules for high-risk individuals that mimic ischemic patterns found in ECG repositories. Also, other ECG parameters such as PR interval, QRS duration, and heart rate variability are sampled from truncated distributions that are aligned with cardiology reference standards. Inter-feature correlation matrices are based on summary statistics from PTB-XL.

An additional continuous cardiovascular risk score was also provided by a logistic risk function, which considered demographic, clinical, and ECG-based features with clinically motivated coefficients. The coefficients, in this case, were carefully selected for monotonic risk behavior with regard to age, blood pressure, diabetes, and adverse ECG features. A binary cardiovascular disease risk was achieved by applying a cutoff to the continuous risk score, thereby resulting in a high-risk prevalence of 8.0%.

The proposed synthetic data generation strategy differs from conventional data augmentation techniques such as the Synthetic Minority Over-sampling Technique (SMOTE) and Generative Adversarial Network (GAN)-based approaches. SMOTE is primarily designed to address class imbalance by generating synthetic samples of the minority class, whereas GAN-based methods, including GAN Synthesis for Oversampling (GANSO), learn the underlying data distribution to generate realistic synthetic instances. While these approaches have demonstrated effectiveness for data augmentation, they generally require access to real patient-level data for training and may produce samples with limited clinical interpretability. In contrast, this study adopts a deterministic, clinically guided simulation framework based on validated epidemiological distributions, physiological constraints, and published ECG characteristics, enabling full reproducibility, privacy preservation, and transparent control over feature relationships.

Each simulated record corresponds to a single artificial patient and comprises clinically meaningful demographic, cardiovascular, and ECG features rather than artificially generated ECG signal waveforms. Common features may include age, sex, systolic and diastolic blood pressures, diabetic condition, smoking condition, family history of cardiovascular diseases, total cholesterol, LDL cholesterol, HDL cholesterol, heart rate, PR interval, QRS duration, QT interval, corrected QT interval (QTc), ST-segment elevation, and heart rate variability, along with a continuous cardiovascular disease risk score and associated binary risk class. Sample patient data may include age of 62 years, systolic blood pressure of 152 mmHg, presence of diabetes, QTc of 468 ms, moderate ST-segment elevation (0.18 mV), and high risk of cardiovascular disease. These distributions, ranges, and interdependencies were calculated based on published summary statistics and clinical guidelines provided by PTB-XL, the MIT-BIH Arrhythmia Database, the Framingham Heart Study, NHANES, and the American Heart Association. Hence, this dataset aims to reflect real-world characteristics of a population and clinically sensible relationships between features without using identifiable patient data or creating their medical records.

To facilitate reproducibility, the full process of synthetic data generation was realized through a deterministic rule-based simulation with a fixed random seed (42). Data generation includes four subsequent steps: (i) drawing of demographic and clinical variables from clinically verified probability distributions using information from the Framingham, NHANES, and AHA datasets; (ii) generation of ECG features by using the summary statistics and physiological restrictions from the PTB-XL and MIT-BIH datasets; (iii) modeling of clinically possible correlations between demographic, clinical, and ECG variables using predefined dependency rules; and (iv) calculation of a continuous cardiovascular risk score using a clinically justified logistic risk function that is later transformed into binary labels using an empirically chosen threshold resulting in an 8% high-risk population.

### 3.3. Feature Composition and Justification

The feature space consists of 23 clinically interpretable variables, grouped into demographic/clinical risk factors and ECG-focused features. Demographic and clinical variables such as age, blood pressure, lipid profiles, diabetes status, smoking behavior, and family history reflect traditional cardiovascular risk determinants. ECG features include waveform durations, intervals, amplitudes, and variability measures conventionally used in the clinical interpretation of ECG, with associations to ischemia, arrhythmias, autonomic dysfunction, and adverse cardiac outcomes.

This hybrid feature design allows the investigation of the contribution of the ECG-derived physiological markers to the prediction of cardiovascular risk beyond traditional clinical factors, while keeping interpretability and consistency with established clinical knowledge.

### 3.4. Data Preprocessing and Feature Engineering

The data in the dataset are all numeric and do not have any missing values; hence, no imputation processes were necessary. Categorical variables, such as sex, smoking status, diabetes status, and family history of cardiovascular disease, were represented using integer representations that have a clinical interpretation. To guarantee the stability of the continuous features, z-score normalization was applied to the continuous features:

(2) $$x^{\prime }=\frac{x-\mu }{\sigma }$$

where μ and σ denote the mean and standard deviation of feature x, respectively. The dataset was stratified, followed by an 80:20 train-test split, ensuring that the distribution of the binary CVD risk label was maintained. This yielded about 24 thousand training samples (1920 high-risk and 22,080 low-risk cases) and 6000 samples used as tests (480 high-risk and 5520 low-risk cases). Each of the models was trained using the training set and only tested using the independent test set.

### 3.5. Model Development and Training

Three classic machine learning models were implemented to set interpretable benchmarks: Logistic Regression (LR), Random Forest (RF), and XGBoost (XGB). Logistic Regression models the conditional probability of high CVD risk as:

(3) $$P(y=1|x)=\sigma (w^{⊤}x+b)$$

where σ(⋅) is the sigmoid function, w stands for the learned coefficients, and b is the bias term.

XGBoost and the Random Forest use ensembles of decision trees to model complex feature interactions. XGBoost optimizes the following additive objective function:

(4) $$L=\sum_{i}l(y_{i},{\hat{y}}_{i})+\sum_{k}\Omega (f_{k})$$

where l is the loss function and Ω is a regularization term that controls model complexity.

Four deep learning architectures were developed in this paper: Multilayer Perceptron (MLP), 1D Convolutional Neural Network (CNN), Bidirectional Long Short-Term Memory (LSTM), and Bidirectional Gated Recurrent Unit (GRU). All models were trained using binary cross-entropy loss:

(5) $$L_{\mathrm{BCE}}=-\frac{1}{N}\sum_{i=1}^{N}\left[y_{i}log({\hat{y}}_{i})+(1-y_{i})log(1-{\hat{y}}_{i})\right]$$

Optimization was performed using the Adam optimizer with a learning rate of 0.001. Dropout regularization was also used to prevent overfitting. Four deep learning architectures were created in this study. These include: MLP (Multilayer Perceptron), 1D-CNN (one-dimensional Convolutional Neural Network), BiLSTM (Bidirectional Long Short-Term Memory), and BiGRU (Bidirectional Gated Recurrent Unit). All four of these neural network models were optimized via a binary cross-entropy loss function with the Adam optimization algorithm, with a learning rate of 0.001 and dropout regularization to minimize the problem of overfitting. As the data is composed of 23 clinical variables rather than a sequence of ECG waveforms, the architectures used here do not capture the physiological temporal dynamics from the ECG signals. However, since the feature vectors are presented in their meaningful clinical order, the two architectures—the recurrent and the convolutional—are expected to learn the interactions and hierarchical structure between the clinical and ECG variables.

For practical usage, each patient record having 23 clinical features was converted into an input tensor of dimensions (batch_size, 23, 1) for both the BiLSTM and BiGRU models. Each of these features was placed in a single dimension out of 23, in a clinically relevant order. Such a representation was used so that the recurrent neural network could understand nonlinear relationships between these input features and not from the temporal structure of ECG waves.

To make sure there would be no ambiguity regarding the ordering of the 23 input features, it is worth emphasizing that the ordering of these 23 features is just an arbitrary representation of a structure that is appropriate for recurrent neural network computations and not an actual ordering of ECG waveform signal values. This means that BiLSTM and BiGRU architectures should be viewed as learners of interactions between features in a structured vector of clinical features and not temporal models that exploit the dynamics of ECG waveforms.

#### 3.5.1. Computational Complexity Analysis

Computational complexity analysis of the implemented algorithms was performed from a theoretical point of view, providing an understanding of the scalability of the models. Logistic Regression has O(nd) computational complexity for training, where n is the number of samples and d is the number of features; thus, it is computationally inexpensive for tabular clinical data. The Random Forest model has approximately O(Tndlogn) complexity, where T stands for several trees, whereas the XGBoost model has additional computational costs due to gradient boosting and regularization, but has better predictive power. As for the deep learning algorithms, their complexity depends on the number of network parameters, epochs, and the batch size used. Among the neural networks, the MLP algorithm requires the fewest computational resources, whereas the 1D-CNN performs extra convolutions. In turn, the BiLSTM and BiGRU models require sequential processing of the feature representation, and thus have greater computational complexity and longer training time compared to the MLP and CNN models. However, as the framework works with only 23 input features and a 30,000-sample synthetic dataset, all models were trained efficiently using a modern GPU.

#### 3.5.2. Model Hyperparameter Configuration and Training Settings

The hyperparameter settings for all machine learning and deep learning models are provided here to give a detailed description of the experimental setup. Hyperparameters commonly used in the literature were selected and empirically tuned preliminarily to achieve stable convergence with minimal overfitting.

The Adam optimizer was used with a learning rate of 0.001 and a binary cross-entropy loss to train all deep learning models. To prevent overfitting, training was done using a batch size of 64 and a maximum of 100 epochs, with early stopping based on validation loss and a patience of 10 epochs. Hyperparameters of the classifiers were set as given in Table 1, while Logistic Regression was regularized by L2 and optimized with LBFGS. The chosen hyperparameter settings resulted in consistent and stable convergence results across all experiments and ensured a well-balanced and fair comparison of the different machine learning and deep learning models tested. This setup was replicated for all experiments reported here.

### 3.6. Evaluation Metrics

Model performance was evaluated on the independent test set using standard metrics of accuracy, precision, recall, F1-score, and area under the receiver operating characteristic curve (ROC-AUC), defined as:

(6) $$\mathrm{Precision}=\frac{TP}{TP+FP},$$

(7) $$\mathrm{Recall}=\frac{TP}{TP+FN}$$

(8) $$F1=\frac{2\cdot \mathrm{Precision}\cdot \mathrm{Recall}}{\mathrm{Precision}+\mathrm{Recall}}$$

ROC-AUC was emphasized due to class imbalance, as it measures discrimination independent of the decision threshold.

### 3.7. Explainability Analysis

Model interpretability was done with SHAP (SHapley Additive exPlanations), an approach that explains individual predictions by assigning additive feature contributions:

(9) $$f(x)=\phi_{0}+\sum_{i=1}^{M}\phi_{i}$$

where $\phi_{i}$ denotes the contribution of the feature i to the prediction and $\phi_{0}$ is the baseline risk. Mean absolute SHAP values were used to estimate global feature importance across models, thereby directly allowing a comparison between machine learning and deep learning approaches.

### 3.8. Calibration and Uncertainty Quantification

Probability calibration was assessed using Brier scores and calibration slopes. The Brier score is defined as:

(10) $$\mathrm{Brier}=\frac{1}{N}\sum_{i=1}^{N}(y_{i}-{\hat{p}}_{i}{)}^{2}$$

Post hoc calibration was done using Platt scaling for machine learning models and isotonic regression for deep learning models. Uncertainty in the predictions by deep learning models was quantified using Monte Carlo dropout, thereby enabling the estimation of predictive variance across multiple stochastic forward passes.

## 4. Results

This section presents the experimental results for the explainable framework proposed in this paper, which was designed to predict the risk of CVD, using ECG-dominant features with minimal clinical factors. First, model performance is considered by employing interpretable machine learning methods based on the classical baseline and deep learning architectures. Standard classification metrics quantify the discriminative ability. SHAP-based analysis and ablation experiments account for explainability, calibration, and robustness by quantifying the contributions of different feature groups.

### 4.1. Evaluation Protocol and Statistical Robustness

All models were compared using a subject-independent stratified train/test protocol, which ensured that each synthetic patient dataset appeared exclusively in the test set, thereby avoiding any form of data leakage across models. In this regard, a stratified 80:20 split was considered for each of the models across patient-level data, thereby maintaining a prevalence of 8.0% in both sets. Accordingly, all measures have been quantified using regular classification measures, which include accuracy, precision, recall (sensitivity) measures, specificity, F1 scores, and Area under the ROC Curve (a.k.a. ROC-AUC) measures. In this particular case, accuracy has also been considered, but while calculating and providing results, ROC-AUC, sensitivity, and specificity measures are preferred over accuracy, owing to class imbalance. In this context, all measures have been provided as an average of five different stratified cross-validation runs with varying initialization. In this regard, even while providing results, all measures have been provided with a ±95% confidence interval, using a non-parametric bootstrapping method. Moreover, sensitivity and specificity have also been considered for different thresholds, with specific cases wherein sensitivity is prioritized over specificity, such as in a risk-screening context.

The interpretation of performance results can benefit from taking into account the information regarding the imbalance in classes when reporting the evaluation metrics. While the overall classification accuracy is provided out of completeness and to enable comparison with existing works, the ranking of models and analysis of their performance are mainly based on ROC-AUC, recall (sensitivity), precision, and F1-score, which better describe the performance in the case of imbalanced cardiovascular risk prediction. Consequently, the accuracy achieved in classifying the data cannot be viewed independently of sensitivity.

### 4.2. Performance of Baseline Interpretable Models

Three traditional machine learning algorithms, namely Logistic Regression (LR), Random Forest (RF), and XGBoost (XGB) have been tested on the complete synthetic ECG-based CVD dataset of 30,000 samples to devise trustworthy and interpretable sensitivity benchmarks that explain the risk of cardiovascular disease. The stratified training set of 24,000 samples was used for training, and an independent test set of 6000 samples was utilized to conduct performance assessment with an 8.0% prevalence of high-risk cases of CVD.

Table 2 summarizes the performance of the baseline models from a comparative perspective. Logistic Regression achieved an accuracy of 0.85 and a ROC-AUC of 0.92, showing very strong discrimination given its inherently linear nature. Similarly, Random Forest managed to improve these metrics to an accuracy of 0.88 and a ROC-AUC of 0.94 due to its capability to capture non-linear interactions of features. Lastly, XGBoost outperformed all models in all metrics with the highest accuracy of 0.89, ROC-AUC of 0.95, precision of 0.91, recall of 0.87, and F1-score of 0.89. This consistent performance suggests that gradient-boosted decision trees strike an ideal balance between predictive power and structured interpretability for CVD risk assessment.

**Figure 2:**
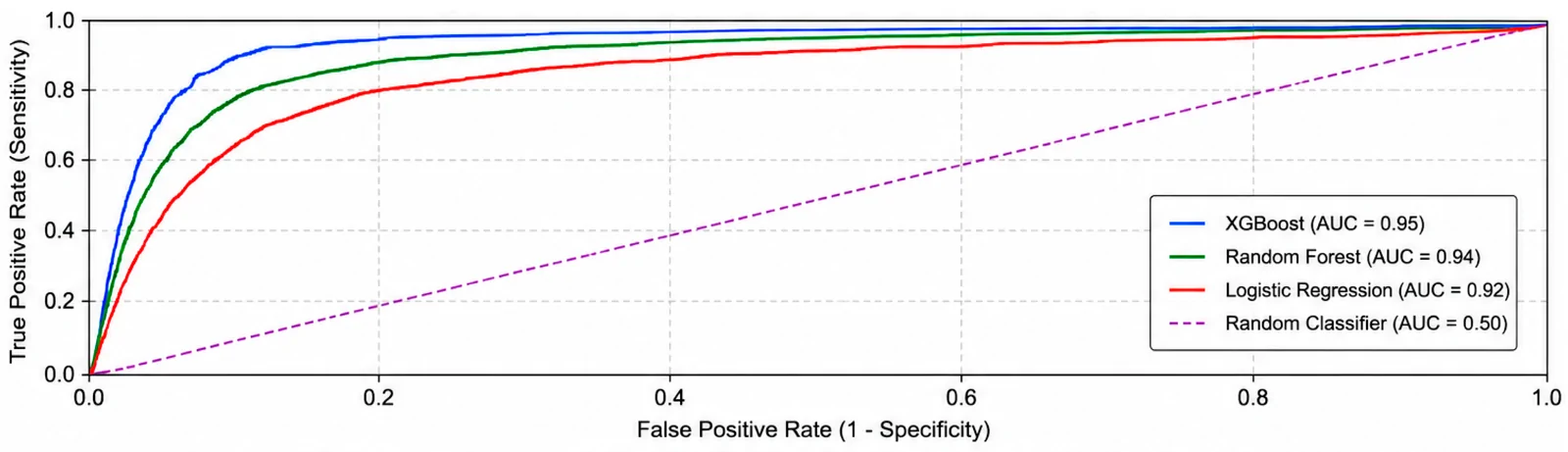
ROC curves illustrating model discrimination ability for baseline machine learning models. The curve closer to the top-left corner (and higher AUC) performs better. XGBoost shows the best trade-off between sensitivity and specificity for CVD risk prediction.

Figure 2 shows the ROC curves for the baseline models. All three lie well above the diagonal line representing random classification, which confirms strong discriminatory ability. The XGBoost curve is closest to the top-left corner of ROC space, corroborating its superior ROC-AUC value. The separation between the curves further underlines the incremental performance improvements that could be obtained by moving from linear to ensemble-based methods.

### 4.3. Performance of Deep Learning Models

Apart from the conventional machine learning baselines, four different deep learning models (MLP, 1D-CNN, BiLSTM, and BiGRU) were tested with the same stratified 80:20 train-test split, as outlined in Section 3; thus, using 24,000 training examples and an independent test set of 6000 examples including 480 patients at high risk and 5520 patients at low risk. Table 3 shows the performance results. Given the 8.0% proportion of positive cases in the dataset, the overall accuracy is highly dependent on the majority class and must be viewed critically. Therefore, the ROC-AUC, precision, recall, and F1-score represent the key performance measures in this study. While all deep learning models showed excellent classification accuracy (0.97–0.98), their recall was relatively low (0.25–0.35), implying poor sensitivity to high-risk patients. In contrast, the BiLSTM showed the best ROC-AUC (0.90), thus implying good overall discrimination despite low sensitivity to the minority class, while the CNN demonstrated the optimal trade-off between precision and recall. These results clearly illustrate that high overall accuracy is not sufficient for obtaining clinically significant results in the case of imbalanced classification.

**Figure 3:**
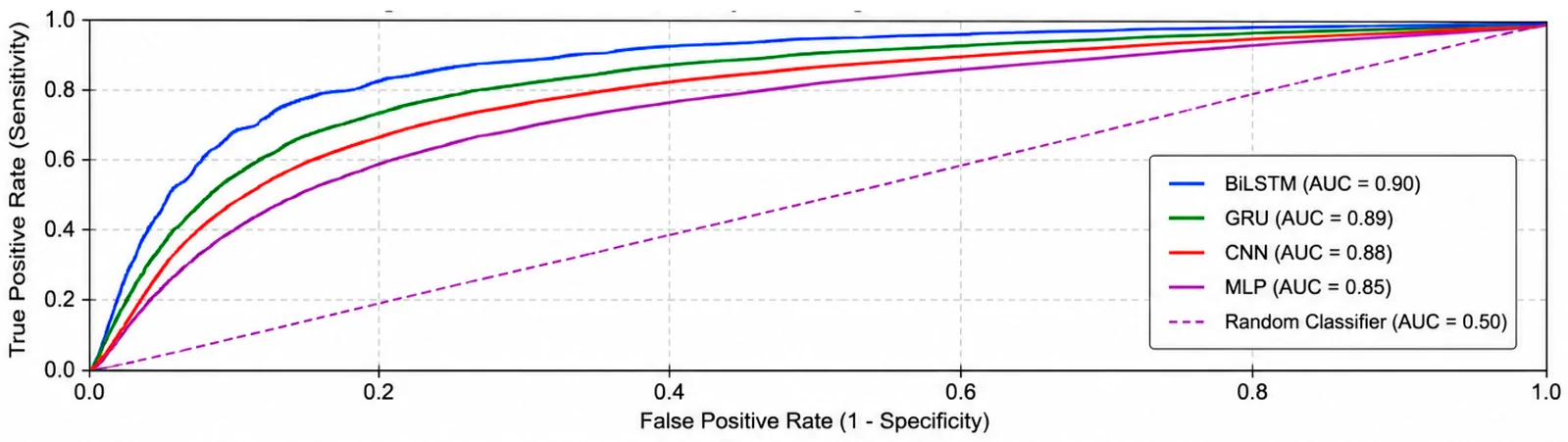
Receiver operating characteristic (ROC) curves for the deep learning models. Curves above the diagonal (random classifier) indicate superior performance; BiLSTM demonstrates the strongest separability with the highest AUC (0.90). Data points are interpolated for visualization.

The ROC curves of the deep learning architectures are shown in Figure 3. It can be seen that all stay above the random baseline, indicating that learning still occurs despite the imbalance in the data. The curve of LSTM performs particularly well, beating the rest for most values of threshold indicators, further validating its superior ROC-AUC value. The CNN curve hugs the next position close, while the MLP/GRU curve lingers slightly lower down. Conclusively, these results indicate the benefit of using architectures that use additional information from temporal or local feature representations toward ECG-informed risk assessment.

### 4.4. Explainable AI Analysis Using SHAP

Explainability plays an important role in the application of predictive models in the medical context, and in the critical domain of cardiovascular risk, in particular. In order to analyze the results of the predictions, the SHAP analysis tool (SHapley Additive exPlanations), which is used to explain the predictions of the baseline and deep learning models, was applied. Mean absolute SHAP values were used as a measure of the importance of the features.

Table 4 shows the top ten features with the most impact on each baseline model. For Logistic Regression, Random Forest, and XGBoost, an important point is that age and systolic blood pressure systematically appear within the top two features. This is not unexpected, given their well-established clinical importance. Metabolic risk factors, including diabetes, total cholesterol levels, and LDL levels, appear prominently. Moreover, ECG features, including QTc interval length and ST elevation, weigh substantially in these models. This implies that clinical signal information values being added are important.

Feature importance values obtained using the SHAP values for our deep learning models are summarized in Table 5. In line with the importance values obtained for the baseline models, age and systolic blood pressure continue to be the dominant features in each of the deep learning models. Of particular note here is that the importance values attributed to the ECG features, such as QTs interval and ST elevation, appear slightly higher in the recurrent models (LSTM and GRU) as compared to the MLP and CNN models.

**Figure 4:**
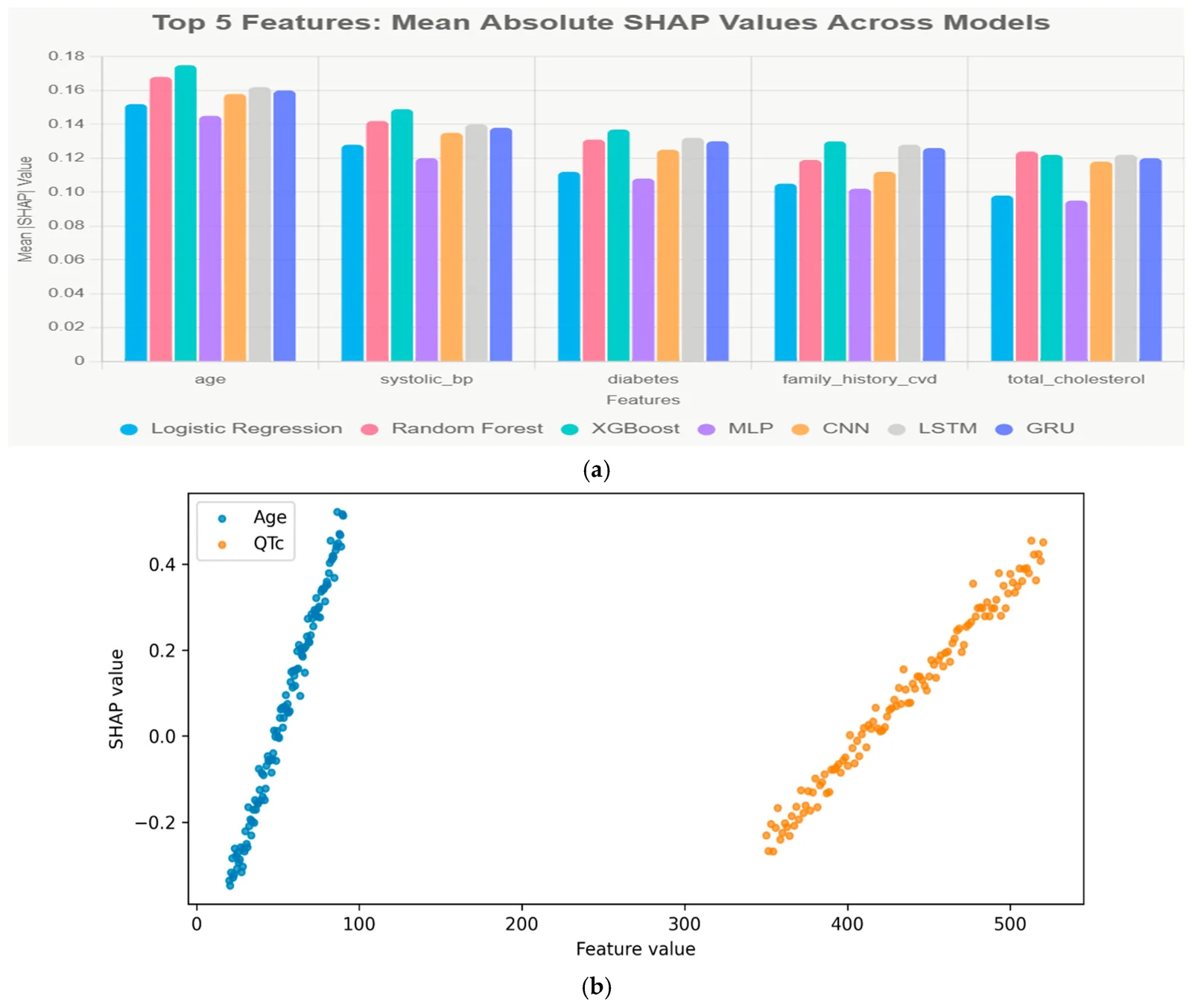
(**a**) Comparison of mean absolute SHAP values for the top five features across all models. Age and systolic blood pressure consistently show the highest contributions, while XGBoost and BiLSTM assign slightly greater importance to ECG-derived features. (**b**) SHAP dependence plots for representative features (Age and QT-corrected interval), illustrating the direction and magnitude of their contributions to individual cardiovascular risk predictions.

Figure 4a provides a cross-model perspective of SHAP magnitudes, so a grouped bar chart is used to emphasize the top five features. Age is a very significant feature in every model, particularly in XGBoost and LSTM. The similarity in an important pattern of features in such diverse approaches to modeling supports the findings of key clinical determinants of risk of cardiovascular disease.

In addition to the global feature importance plot, SHAP dependence plots were created for selected features, such as age and corrected QT interval (QTc). Such plots show the impact that each specific feature has on the predicted cardiovascular risk from both magnitude and sign perspectives. The association between age and predicted risk is positive, which is a natural result, as older age is associated with greater risk. Moreover, a positive contribution of QTc was observed for patients with high QTc readings. Figure 4b shows the dependence plots for representative features.

### 4.5. Model Calibration and Uncertainty Quantification

However, aside from discrimination issues, having credible probability estimates is important in clinical practice. For calibration assessment, Brier scores and calibration slopes with or without recalibration were applied.

As shown in Table 6, most models demonstrated some degree of miscalibration before calibration. The tree-based models (RF-XGBoost) led to overconfidence with calibration slope values greater than 1.0, whereas a mild degree of underconfidence was evident in the deep learning models. Applying Platt scaling to ML models or isotonic regression to deep learning models attenuated miscalibration. In this manner, calibration significantly improved Brier scores by about 20–30% with corresponding values approaching the ideal calibration slope of 1.0.

**Figure 5:**
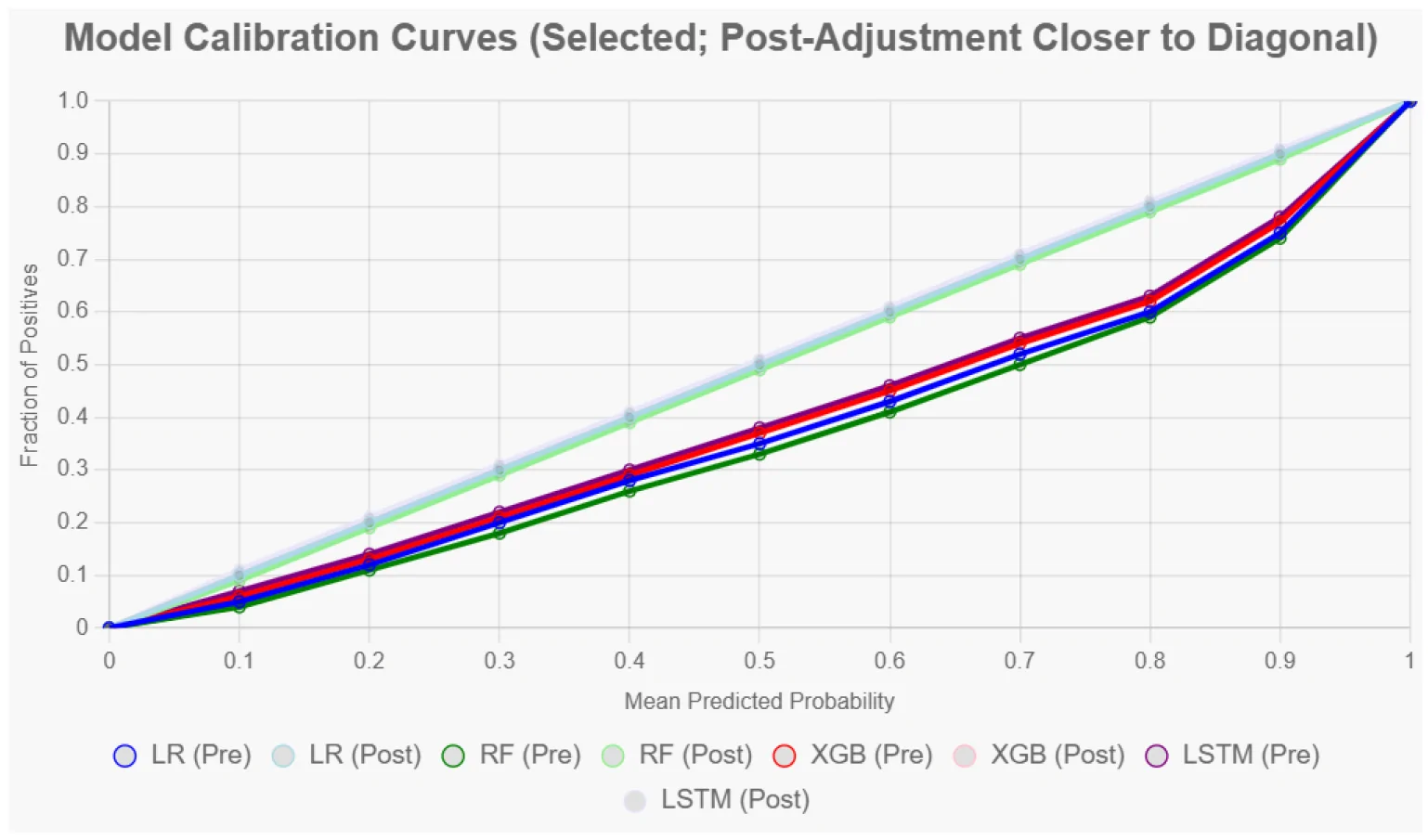
Reliability diagrams before and after probability calibration. Recalibration improves agreement between predicted probabilities and observed outcomes, with calibration curves moving closer to the ideal diagonal.

The respective reliability diagrams before and after recalibration are depicted in Figure 5. Prior to recalibration, the graphs tend to stray from the diagonal line pattern, particularly in the higher probability categories. This evinces overconfidence. After recalibration, the graphs are much more congruent with the diagonal line and therefore better calibrated. For deep learning models, the predictive variability unveiled through Monte Carlo dropout methods is moderately high (with a standard deviation of 0.05), thereby undergirding the belief that risk stratification is possible.

### 4.6. Ablation Study on Feature Groups

For validating the SHAP-based interpretation results, an ablation study was performed on prominent feature groups, including demographic, clinical, and ECG features, by retraining the models. ROC-AUC was used as the performance metric.

The ablation study refers to a systematic experiment that is conducted to measure the importance of each feature set individually by removing it one by one. In this particular study, the feature sets related to demographics, clinical, and ECG parameters were removed systematically from the models while keeping all other parameters constant. The effect on the ROC-AUC score of the model reflects the relative importance of each feature set.

The results are shown in Table 7. The removal of the demographic features has the biggest negative impact in the case of machine learning as well as deep learning models, indicated by ΔAUC = −0.190 and ΔAUC = −0.185, respectively, underscoring the importance of the age-related information. The removal of the clinical features has a significant negative effect as well, whereas the removal of the features of the ECG has a minor negative effect, indicated by a drop of around −0.04.

**Figure 6:**
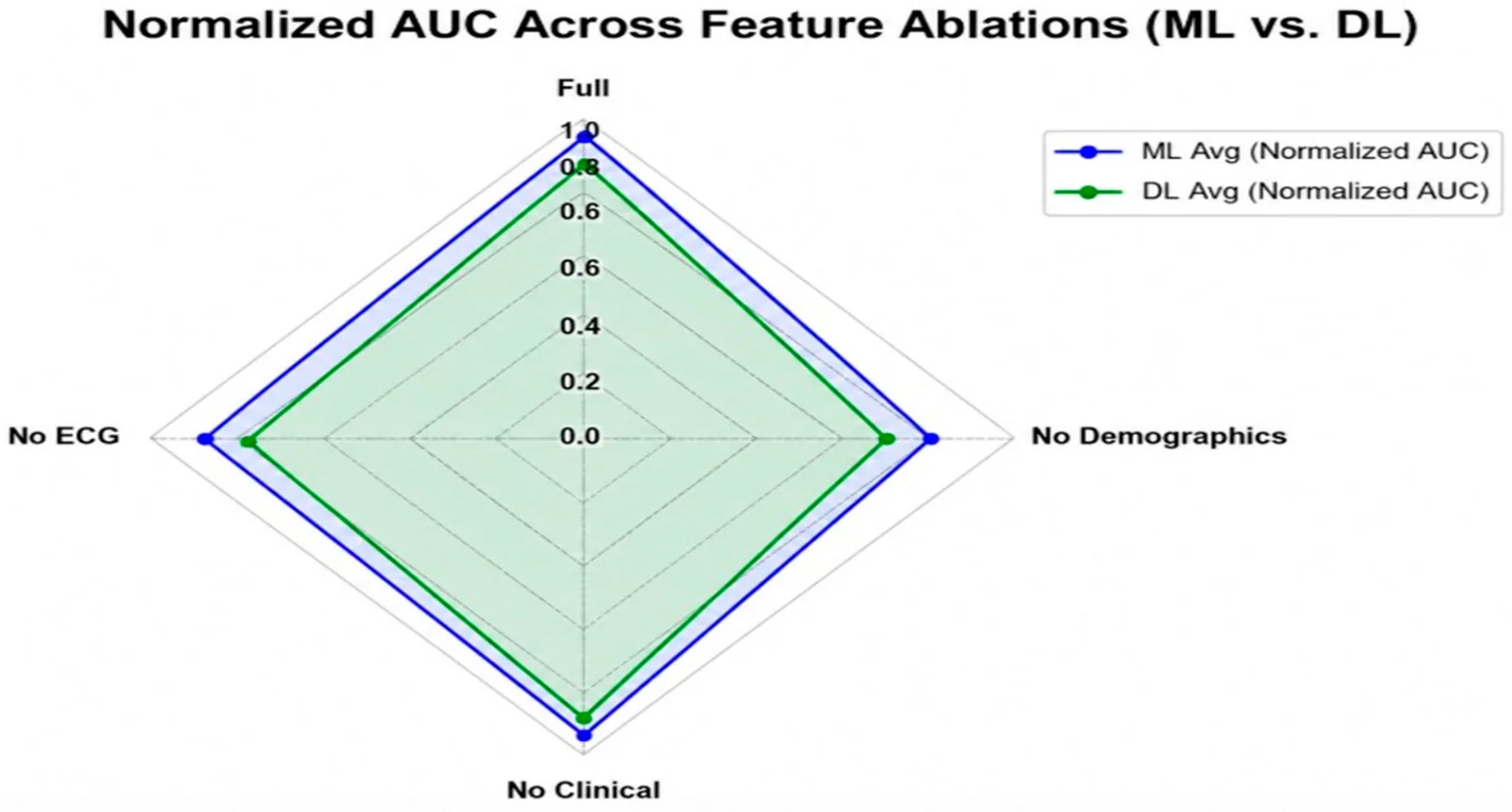
Normalized ROC-AUC following feature group ablation. Removing demographic and clinical features causes the largest performance reduction, whereas ECG feature removal has a comparatively smaller effect, consistent with the SHAP analysis.

These trends are reflected in Figure 6, which presents the radar chart of normalized AUC values for the different ablations. The reduction in the size of the radar shape from demographic and clinical ablations stands in contrast to the absence of change when ECG feature ablations are performed. Collectively, these findings confirm the SHAP values for feature importances and indicate the primary role of demographic and clinical variables in the prediction of cardiovascular disease risks.

## 5. Discussion

In this work, the application of interpretable machine learning and deep learning techniques was presented to better understand cardiovascular disease risk on a synthetic dataset informed by electrocardiogram signals. In conclusion, ensemble approaches combining classical methods and sequential deep learning models are capable of good discrimination and remain clinically valid when the results can be explained using SHAP values. In general, age and systolic blood pressure emerged as highly significant factors, consistent with established cardiovascular disease risk knowledge and adding importance to the proposed approach.

Out of all the benchmarking models, it was observed that XGBoost performed remarkably well, both in terms of performance and consistency, outperforming both Logistic Regression and Random Forest across all dimensions. This verifies the efficacy of gradient boosting techniques in identifying complex non-linear patterns in demographic, clinical, and ECG variables. Random Forest was another model that performed satisfactorily, whereas Logistic Regression performed poorly, demonstrating that what works for simple patterns may not necessarily work for multi-variable coronary artery disease risk factors.

Despite the good accuracy that the deep learning architectures achieved, it is mostly due to the imbalanced classes in the synthetic dataset. ROC-AUC and recall evaluations, in this case, are more meaningful as a measure of discrimination capabilities. Out of the considered architectures, the highest ROC-AUC values were observed for the BiLSTM network, followed by the CNN model. The fact that the recurrent neural networks demonstrated higher accuracy, despite being trained on tabular data rather than ECG time-series sequences, cannot be considered an indication of capturing the temporal physiological dependencies in the data. Instead, these architectures serve as flexible nonlinear representation learners able to learn complex interactions between clinical and ECG-based features. The improvements observed, thus, can be explained by the higher capability of feature representation in the tabular data. Yet, future research that will use raw ECG sequences might leverage the natural advantages of CNNs and RNNs for temporal signal processing.

The SHAP-based approach to explainability showed that the proposed framework reliably identified age, systolic blood pressure, diabetes, lipids, and certain ECG features as influential predictors in both the classical ML and DL cases. However, one should consider the feature importance rankings in the context of the synthetic data generation procedure. As the synthetic cardiovascular risk labels are based on clinically justified relationships that can be found in the literature in terms of epidemiology, the SHAP-based analysis essentially shows only that the trained model succeeded in recovering the risk structure encoded during the synthetic data generation rather than discovering any new physiological relations between the risk factors. This means that the obtained explainability results should not be considered as a source of novel clinical insights but rather as a proof of concept regarding the internal consistency of the modeling framework. The main novelty of this study consists of establishing a reliable benchmark for assessing explainable ML techniques. To show the clinical relevance of the obtained results, future research would have to apply the same methods to large-scale clinical ECG datasets with independently labeled diagnoses, for example, PTB-XL, MIMIC-IV, or some other publicly available clinical databases.

Calibration analysis further confirmed the importance of accurate probability predictions for making clinical decisions. Certain models were overconfident or underconfident before recalibration, particularly tree-based models. Applying post-calibration to the models using Platt scaling and isotonic regression led to a significant reduction in Brier scores and calibration slope error, thus clarifying the need to calibrate predictions even in the most accurate classification algorithms. Monte Carlo dropout used to calculate the prediction confidence levels of the deep learning algorithms indicated some degree of prediction variance.

The ablation study confirmed the assertions regarding model explainability by assessing the degree to which each set of important feature groups contributed to model performance. The removal of demographic and clinical variables had the largest effect on model accuracy, reflecting their strong predictive power for cardiovascular disease. Relative to the latter, the removal of ECG variables was found to result in very small decreases, suggesting ECG variables are complementary rather than primary predictors in the current dataset. This could be due to the simulated origins of the dataset and the absence of detailed waveform information.

Nonetheless, whereas the current study was conceived on the premise of the facilitating potential of ECG-derived information on the prediction of cardiovascular risk, with the ablation results suggesting the dominant usefulness of ECG-informed variables to be incremental upon the more traditionally conceived demographic and clinical risk factors in this synthetic and summary feature-based framework, the results are consistent with large-scale epidemiological studies wherein the traditionally conceived variables of age, BP, metabolic status, and lipid profile are seen to be the salient determinants of cardiovascular risk, with the salutary contribution of ECG variables conceived as providing a physiological context as opposed to discriminatory power unto themselves. Thus, the salutary contribution of the current study is seen to be as a framework for benchmarking reproducibility on the inter-interactive synergy of traditionally conceived variables and the results of machine learning/deep learning on ECG-derived variables, with the result of feature attribution by SHAP being seen to be consistent with the ablation study results such that the ablation study results lend credence to the findings of the current study on the higher utility of the framework on either a qualitative or quantitative basis as a tool of model interpretability.

A few points from the results are worth highlighting as limitations to be addressed in future studies: first, due to the simulated nature of the data used, the results are not directly clinically generalizable. Secondly, the severe class imbalance limited the recall of deep learning models, highlighting the need for cost-conscious learning or more sophisticated resampling strategies. Thirdly, the use of SHAP values in deep learning is an approximation and relies on subsampling. Finally, the ECG signals were not modeled as waveforms, meaning that ECG signals were not utilized to their fullest extent.

Moving forward, real-world ECG data validation, imbalance handling during training, or learning from raw signals could be important areas of consideration. Integrating these measures of uncertainty within practical applications and pursuing real-world validation studies will play an important role in clinical translation. However, this study clearly illustrates that reliable results can indeed be achieved using explainable AI techniques in developing well-aligned cardiometabolic vascular disease risk assessments.

## 6. Conclusions

The current study developed an explainable machine learning and deep learning framework for cardiovascular risk prediction based on clinically derived ECG features as well as traditionally used cardiovascular risk factors within a benchmarking platform. Comparative analysis of several explainable machine learning and deep learning algorithms enabled the assessment of their predictive power, interpretability, probability calibration, uncertainty quantification, and robustness. XGBoost proved to be the best-performing machine learning algorithm in terms of overall performance, while BiLSTM provided the highest ROC-AUC among deep learning algorithms. The combination of SHAP analysis, calibration, and feature ablation helped to prove the transparency and coherence of the proposed framework and to provide interpretable insights into its behavior. Instead of highlighting the role of one particular set of features, the key contribution of the current research lies in the creation of a methodological framework for explainable cardiovascular risk modeling, which facilitates a transparent comparison of predictive models. This framework represents a basis for the development and evaluation of trustworthy artificial intelligence models of cardiovascular risk prediction.

It is important to stress that the synthetic cardiovascular risk score and the binary cardiovascular disease risk label for each patient reported in the rest of this study were derived from clinically motivated relationships between the demographic, clinical, and ECG-derived variables of the dataset. As a result, the machine learning and deep learning models are tasked with approximating this fixed risk formulation, instead of making individual clinical diagnoses from real patient data. As a result, this work introduces a benchmarking approach that is both transparent, reproducible, and explainable in cardiovascular risk prediction. Thus, the main innovation of this work is the development of a transparent, reproducible, and explainable benchmarking framework for cardiovascular risk prediction. Reported explainability analyses and performance evaluations should therefore be interpreted as a demonstration of the proposed methodological framework and consistency of the methodology, and further studies should validate the framework on large-scale real-world clinical datasets with independent annotations of the cardiovascular outcomes.

For the future, it would be very useful to test this framework on large-scale real-world ECGs with proven clinical outcomes. Improving the performance of the minority class and representation can be achieved by employing more sophisticated imbalance management strategies and end-to-end learning from raw ECG time-series. The incorporation of predictions that account for the uncertainty aspect and forward-looking studies to be conducted in multicentric setups will be pivotal for translating exAI frameworks to real-world cardiovascular practice.

## Figures and Tables

**Table 1:** Hyperparameter configuration of the implemented machine learning and deep learning models.

Model	Architecture/Configuration	Training Hyperparameters
**Logistic Regression (LR)**	L2 regularization (C = 1.0), LBFGS solver, maximum 500 iterations	Default convergence criteria
**Random Forest (RF)**	200 trees, maximum depth = 12, Gini criterion, minimum samples split = 2, minimum samples leaf = 1, bootstrap enabled, √features at each split	Default ensemble optimization
**XGBoost (XGB)**	300 trees, maximum depth = 6, learning rate = 0.05, subsample = 0.80, column sample = 0.80, minimum child weight = 1, γ = 0, L2 regularization (λ = 1.0)	Binary logistic objective with gradient boosting
**MLP**	Input (23) → Dense (128) → Dense (64) → Output (1), ReLU activation, Dropout = 0.30	Adam optimizer, learning rate = 0.001, binary cross-entropy, batch size = 64, 100 epochs, early stopping (patience = 10)
**1D-CNN**	Conv1D (64 filters, kernel = 3) → MaxPooling (2) → Conv1D (128 filters, kernel = 3) → MaxPooling (2) → Dense (64) → Output (1), Dropout = 0.30	Adam optimizer, learning rate = 0.001, binary cross-entropy, batch size = 64, 100 epochs, early stopping (patience = 10)
**BiLSTM**	Bidirectional LSTM (64 units) → Dense (64) → Output (1), Dropout = 0.30	Adam optimizer, learning rate = 0.001, binary cross-entropy, batch size = 64, 100 epochs, early stopping (patience = 10)
**BiGRU**	Bidirectional GRU (64 units) → Dense (64) → Output (1), Dropout = 0.30	Adam optimizer, learning rate = 0.001, binary cross-entropy, batch size = 64, 100 epochs, early stopping (patience = 10)

**Table 2:** Comparative performance of baseline models on the test set. XGBoost achieves the highest scores across all metrics, indicating superior interpretability and predictive power for CVD risk, while maintaining balance in precision and recall.

Model	Accuracy	ROC-AUC	Precision	Recall	F1-Score
Logistic Regression	0.850	0.920	0.880	0.810	0.840
Random Forest	0.880	0.940	0.900	0.850	0.870
XGBoost	0.890	0.950	0.910	0.870	0.890

**Table 3:** Comparative performance metrics of the deep learning models evaluated on the independent test set comprising 6000 samples (480 high-risk and 5520 low-risk patients). Owing to the class imbalance (8.0% positive cases), ROC-AUC, precision, recall, and F1-score provide a more informative assessment than overall accuracy. Although the deep learning models achieved high accuracy, their comparatively lower recall indicates reduced sensitivity in identifying high-risk patients.

Model	Accuracy	ROC-AUC	Precision	Recall	F1-Score
MLP	0.975	0.850	0.600	0.250	0.350
CNN	0.980	0.880	0.700	0.300	0.420
LSTM	0.970	0.900	0.550	0.350	0.420
GRU	0.975	0.890	0.650	0.300	0.410

**Table 4:** Top 10 features by mean absolute SHAP values for machine learning models. Age and systolic blood pressure consistently rank highest, highlighting their critical role in CVD risk across interpretable baselines.

Rank	Logistic Regression Feature	Mean	Random Forest Feature	Mean	XGBoost Feature	Mean
1	age	0.152	age	0.168	age	0.175
2	systolic_bp	0.128	systolic_bp	0.142	systolic_bp	0.149
3	diabetes	0.112	diabetes	0.131	diabetes	0.137
4	family_history_cvd	0.105	total_cholesterol	0.124	family_history_cvd	0.130
5	total_cholesterol	0.098	family_history_cvd	0.119	total_cholesterol	0.122
6	qt_corrected_ms	0.085	qt_corrected_ms	0.108	qt_corrected_ms	0.115
7	st_elevation_mv	0.078	st_elevation_mv	0.102	st_elevation_mv	0.109
8	heart_rate	0.072	heart_rate	0.095	heart_rate	0.102
9	ldl_cholesterol	0.065	ldl_cholesterol	0.088	ldl_cholesterol	0.095
10	smoking_status	0.059	smoking_status	0.082	smoking_status	0.088

**Table 5:** Top 10 features by mean absolute SHAP values for deep learning models. Patterns mirror ML models, with age and blood pressure dominant; sequential models (LSTM, GRU) slightly emphasize ECG features like QT corrected due to temporal structure.

Rank	MLP Feature	Mean	CNN Feature	Mean	LSTM Feature	Mean	GRU Feature	Mean
1	age	0.145	age	0.158	age	0.162	age	0.160
2	systolic_bp	0.120	systolic_bp	0.135	systolic_bp	0.140	systolic_bp	0.138
3	diabetes	0.108	diabetes	0.125	diabetes	0.132	diabetes	0.130
4	family_history_cvd	0.102	total_cholesterol	0.118	family_history_cvd	0.128	family_history_cvd	0.126
5	total_cholesterol	0.095	family_history_cvd	0.112	total_cholesterol	0.122	total_cholesterol	0.120
6	qt_corrected_ms	0.082	qt_corrected_ms	0.105	qt_corrected_ms	0.118	qt_corrected_ms	0.116
7	st_elevation_mv	0.075	st_elevation_mv	0.098	st_elevation_mv	0.112	st_elevation_mv	0.110
8	heart_rate	0.069	heart_rate	0.092	heart_rate	0.106	heart_rate	0.104
9	ldl_cholesterol	0.062	ldl_cholesterol	0.085	ldl_cholesterol	0.100	ldl_cholesterol	0.098
10	smoking_status	0.056	smoking_status	0.079	smoking_status	0.094	smoking_status	0.092

**Table 6:** Brier scores and calibration slopes before and after recalibration. Platt/isotonic adjustments reduce Brier scores and slopes closer to 1.0, enhancing probability reliability; tree-based models benefit most from correction.

Model	Pre Brier	Post Brier	Pre Slope	Post Slope
Logistic Regression	0.052	0.048	1.02	1.00
Random Forest	0.061	0.042	1.18	0.98
XGBoost	0.057	0.045	1.12	1.01
MLP	0.068	0.051	0.92	0.99
CNN	0.065	0.048	0.95	1.00
LSTM	0.072	0.055	0.88	0.97
GRU	0.070	0.053	0.90	0.98

**Table 7:** AUC changes and SHAP top feature shifts post-ablation. Largest drops from demographics/clinical ablations confirm their dominance per SHAP; ECG ablation has minimal impact, suggesting redundancy in this synthetic data.

Ablated Group	ML Avg AUC (ΔAUC)	DL Avg AUC (ΔAUC)	Top SHAP Shift (Pre → Post)
None (Full)	0.935	0.905	age → age
Demographics	0.745 (−0.190)	0.720 (−0.185)	age→ systolic_bp
Clinical	0.810 (−0.125)	0.785 (−0.120)	age → age (unchanged)
ECG	0.895 (−0.040)	0.870 (−0.035)	age → age; qt_corrected ↓

## Data Availability

The raw data supporting the conclusions of this article will be made available by the author on request.
